# An industry survey of the composition and variability of soybean gums and soapstocks across US soybean processing plants

**DOI:** 10.1093/jas/skae378

**Published:** 2024-12-12

**Authors:** Katelyn N Gaffield, Robert D Goodband, Joel M DeRouchey, Mike D Tokach, Jason C Woodworth, Gordon Denny, Paul Smolen, Carmen Slipher, Hari B Krishnan, Jordan T Gebhardt

**Affiliations:** Department of Animal Sciences and Industry, College of Agriculture, Kansas State University, Manhattan, KS, USA 66506-0201; Department of Animal Sciences and Industry, College of Agriculture, Kansas State University, Manhattan, KS, USA 66506-0201; Department of Animal Sciences and Industry, College of Agriculture, Kansas State University, Manhattan, KS, USA 66506-0201; Department of Animal Sciences and Industry, College of Agriculture, Kansas State University, Manhattan, KS, USA 66506-0201; Department of Animal Sciences and Industry, College of Agriculture, Kansas State University, Manhattan, KS, USA 66506-0201; Gordon Denny, LLC, Thornton, CO, USA 80602; Agri Networks Management, Old Saybrook, CT, USA 06475; Bunge North America, Chesterfield, MO, USA 63017; Agricultural Research Service, U.S. Department of Agriculture and Divisions of Plant Sciences and Animal Sciences, University of Missouri, Columbia, MO, USA 65211; Department of Diagnostic Medicine/Pathobiology, College of Veterinary Medicine, Kansas State University, Manhattan, KS, USA 66506-0201

**Keywords:** gums, lipid quality, soapstocks, soybean meal quality, soybean by-products, trypsin inhibitor units

## Abstract

Depending on the soybean processing plant, gums and soapstocks may be added back to soybean meal during soybean processing. Despite potential effects on soybean meal quality, there is limited information available on the composition and variation in soybean by-products and the resulting soybean meal if by-products are added back during processing. A total of 36 soybean by-product samples from 14 plants across 8 different companies were examined in an industry survey evaluating the composition and variation of soybean gums and soapstocks across the United States. All soybean processing plants in the study produced at least 1 of the 2 by-products: soybean gums or soybean soapstocks. Soybean by-product and soybean meal samples were collected within 2 different timeframes: May to July 2023 and October to November 2023. The individual plants surveyed constitute approximately 30% of total US soybean meal production, with 8 participating companies representing 80% of the total US soybean meal production. By-products were analyzed for lipid quality criteria including moisture, fat by acid hydrolysis, fatty acid analysis, and oxidation markers. Soybean meal samples were submitted for analysis of the proximate composition, neutral detergent fiber, Ca, P, and trypsin inhibitor units. Soybean gums had a greater (*P* ≤ 0.05) percentage of acid-hydrolyzed fat and p-Anisidine value compared to soybean soapstocks. Soybean soapstocks tended to have a greater (*P* = 0.085) percentage of moisture and volatile matter as well as an increased (*P* = 0.052) concentration of insoluble impurities compared with soybean gums. Most notably, there was considerable variation in the composition of by-product samples among processing plants indicating differences in processing procedures or incoming soybean quality. Soybean meal containing added soybean by-products had 61% greater (*P* < 0.05) ether extract than soybean meal samples without added soybean by-products on a dry matter basis, but there was no difference (*P* > 0.10) in crude protein. Furthermore, trypsin inhibitor units varied considerably among plants with values ranging from 1.45 to 9.26 TIU/mg of seed powder, regardless of by-product inclusion. These results provide information on the composition and variation in soybean by-products across various processing plants; however, further information is still needed to evaluate their subsequent effects on livestock diets.

## Teaser

There was considerable variation in soybean by-product composition among processing plants. Ether extract percentage of soybean meal was increased when soybean by-products were added back to soybean meal during processing, but crude protein was unaffected.

## Introduction

**Figure 1.**
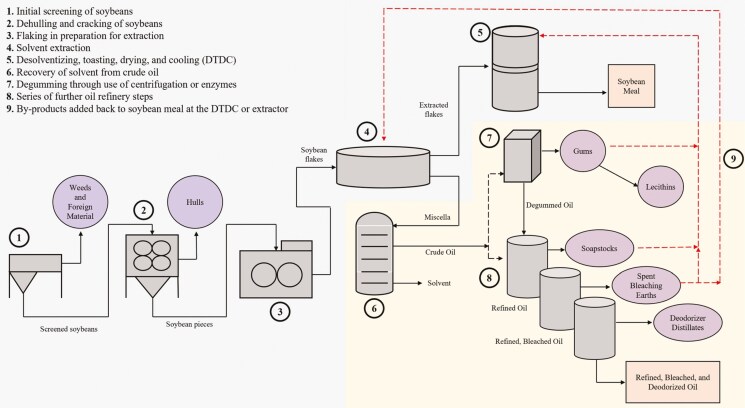
Soybean processing by-product production throughout the soybean crushing and oil refining process. Reprinted from Gaffield, K. N., R. D. Goodband, J. M. DeRouchey, M. D. Tokach, J. C. Woodworth, G. Denny, and J. T. Gebhardt. 2024. A review of soybean processing by-products and their use in swine and poultry diets. Trans. Anim. Sci. 8:1-8. doi:10.1093/tas/txae063.

Soybean meal is an important, high-quality protein source frequently used in livestock diets. In 2021, approximately 34 million metric tons of soybean meal were used in animal feeds, with 78% in poultry and swine diets (United Soybean Board, 2021). Therefore, understanding soybean meal composition, quality, and nutritive value is a crucial area of focus in monogastric nutrition. Despite the focus on soybean meal research, there has been limited attention paid to the composition and nutritional value of soybean processing by-products such as soybean gums and soapstocks. Although they are by-products of soybean oil refining, soybean gums and soapstocks have the potential to affect soybean meal quality. Soybean processors must find a market for or method of utilizing each by-product produced throughout the process, and one of the easiest and most affordable methods of managing soybean gums and soapstocks is adding them back to soybean meal during processing (Gerde et al., 2020). This is most common when an oil refinery is adjacent to a soybean crushing facility (Figure 1; reprinted from Gaffield et al., 2024).

Soybean gums are produced through the degumming step of oil refining in an effort to remove phosphatides from oil, while soybean soapstocks are produced during caustic refining which removes any remaining phosphatides and neutralizes free fatty acids (Erickson, 1995a, 1995b). If added back to soybean meal during processing, these by-products will be transferred to the desolventizing, toasting, drying, and cooling steps of production to be dried along with soybean meal. These by-products have been estimated to contain approximately 35% crude oil (Erickson, 1995a, 1995b). Therefore, adding these by-products back to soybean meal may have an effect on its quality.

The challenge with understanding how gums and soapstocks will affect soybean meal quality is the currently limited information on the composition of soybean by-products themselves. Multiple factors can impact the composition of soybean by-products including initial soybean quality, degumming procedures, caustic refining procedures, age of machinery, and facility management (Erickson, 1995a, 1995b; O’Brien, 2008). These factors likely lead to the composition of by-products varying across soybean processing plants. To fully understand the potential implications on growth performance and health of non-ruminant animals fed soybean meal containing added by-products, the industry first needs a better understanding of the composition and consistency of the soybean gums and soapstocks. Therefore, the objective of this study was to investigate the composition and variability of soybean gums and soapstocks across US soybean processing plants and their impact on subsequent soybean meal quality through an industry survey.

## Materials and Methods

A total of 36 soybean by-product samples from 14 soybean processing plants across 8 different companies were used in an industry survey. The individual plants surveyed constitute approximately 30% of total US soybean meal production, while the 8 participating companies represent 80% of the total US soybean meal production. All soybean processing plants within the study produced at least 1 of the 2 by-products: soybean gums or soybean soapstocks. Soybean by-product and soybean meal samples were collected in 2 different timeframes within the survey: May to July 2023 and October to November 2023. At each timeframe, by-product samples included 12 soybean gum and 6 soybean soapstock samples. Furthermore, a total of 26 soybean meal samples were collected. At each timeframe, 7 soybean meal samples contained added by-products, and 6 samples did not contain added by-products. It is important to note that 2 plants did not submit samples by the submission deadlines for this study; therefore, they will only have data presented during one timeframe.

Plants received six 500 mL bottles (3 bottles for each by-product; Nalgene lab quality amber HDPE bottles, Thermo Scientific, Waltham, MA) and 2 207 mL sampling bags (Whirl-Pak, Nasco, Ft. Atkinson, WI). Upon receipt of sampling packages, an employee from each soybean plant collected the by-product samples applicable to their production system (soybean gums and/or soapstocks). Simultaneously, a soybean meal sample was collected from each plant. Information regarding the date of collection, employee name and contact, and if soybean processing by-products had been added back to soybean meal during the time of collection were recorded. Once collected, samples were immediately shipped overnight to Kansas State University to be prepared for analysis. All samples were stored at 4 °C until analysis was performed.

### Chemical analysis

Samples of soybean gums and soapstocks were submitted for lipid quality analysis. Each by-product sample was homogenized at the laboratory prior to being analyzed. For both timeframes, the following criteria were measured in duplicate: moisture and volatile matter by hot plate (Method Ca 2b-38; AOCS, 2017 and Method Ba 2a-38; AOCS, 2022), insoluble impurities (Method Ca 3a-46; AOCS, 2021), unsaponifiable matter (Method Ca 6a-40; AOCS, 2017), P (Methods 984.27, 927.02, 985.01, and 965.17; AOAC, 1996), p-Anisidine value (Method Cd 18-90; AOCS, 2017), fat by acid hydrolysis (Method 954.02; AOAC, 1977), fatty acid profile (Methods Ce 2-66 and Ce 1b-89; AOCS, 2017), and free fatty acids (Method 940.28; AOAC, 2012 and Method Ca 5a-40; AOCS, 2017).

Soybean meal samples were ground and analyzed in duplicate for Ca and P (Method 985.01 A, B, and D; Method 942.05; AOAC, 2006). Soybean meal samples were also analyzed in duplicate for proximate composition including dry matter (DM; Method 930.15; AOAC, 1999), crude protein (Method 990.03; AOAC, 2002), ether extract (Method 2003.05; AOAC, 2006), crude fiber (Method Ba 6a-05; AOCS, 2017), and ash (Method 942.05; AOAC, 2005) as well as neutral detergent fiber using Ankom technology (Method 2001.11; AOAC, 2005). A sample of each soybean meal was submitted for analysis of trypsin inhibitor units (TIU) in triplicate utilizing procedures outlined by Kim and Krishnan (2023).

### Statistical analysis

Data were analyzed as an ANOVA using the lmer function from the lme4 package in R (version 2023.12.0 (2023-12-17), R Foundation for Statistical Computing, Vienna, Austria). For by-product analysis, soybean by-product type (soybean gums or soybean soapstocks) served as the fixed effect. For soybean meal analysis, soybean meal by-product inclusion (no by-products added or by-products added) served as the fixed effect. The sample was included as a random effect to account for duplicate analysis. All results were considered significant with *P* ≤ 0.05 and marginally significant with *P* ≤ 0.10. Descriptive statistics were included to show the simple means of each analytical criterion within by-product or soybean meal type and timeframe of sample collection. Additionally, the range was included which represented minimum and maximum values for each analytical criterion within the soybean by-product or soybean meal type and timeframe of sample collection. The range was based on an individual analysis and does not represent the values of a sample analyzed in duplicate.

## Results

**Figure 2.**
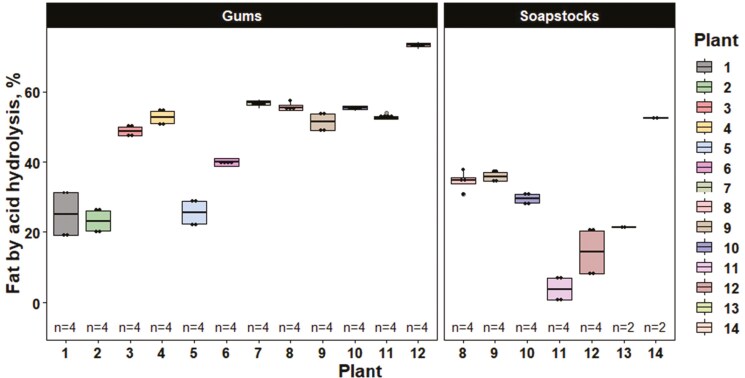
Fat by acid hydrolysis of soybean by-product samples by soybean processing plant and soybean by-product type (as-is). Individual boxplots are presented for each soybean processing plant where each data point represents a sample analyzed in singlet. The line within the boxplot represents the median, the lower and upper bounds represent the 25th and 75th percentiles, respectively, the lower and upper whiskers represent the minimum and maximum data points within 1.5 times the interquartile range, and individual data points outside the whiskers represent points exceeding 1.5 times the interquartile ranges.

**Figure 3.**
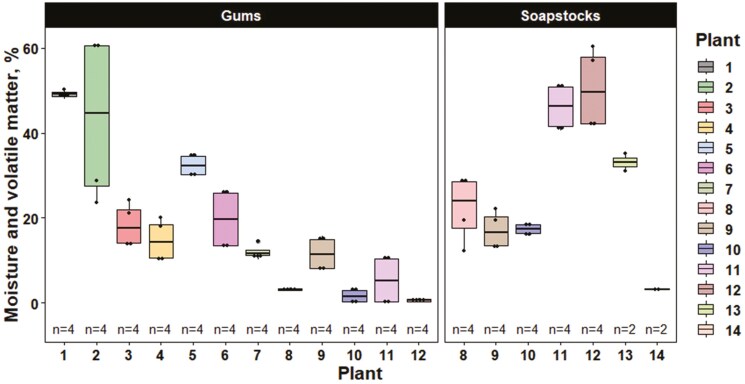
Moisture and volatile matter of soybean by-product samples by soybean processing plant and soybean by-product type (as-is). Individual boxplots are presented for each soybean processing plant where each data point represents a sample analyzed in singlet. The line within the boxplot represents the median, the lower and upper bounds represent the 25th and 75th percentiles, respectively, the lower and upper whiskers represent the minimum and maximum data points within 1.5 times the interquartile range, and individual data points outside the whiskers represent points exceeding 1.5 times the interquartile ranges.

**Figure 4.**
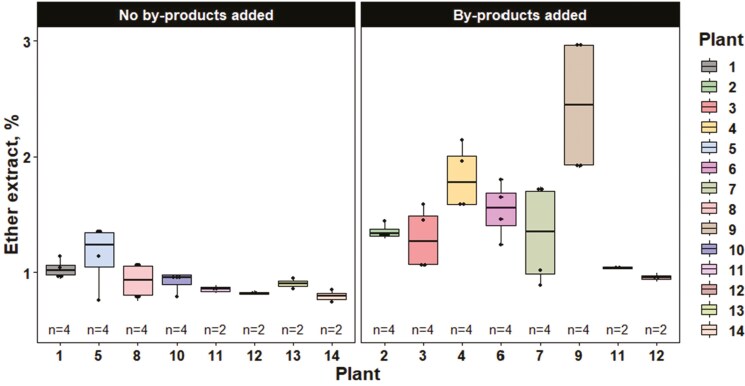
Ether extract of soybean meal samples by soybean processing plant and soybean by-product inclusion (as-is). Individual boxplots are presented for each soybean processing plant where each data point represents a sample analyzed in singlet. The line within the boxplot represents the median, the lower and upper bounds represent the 25th and 75th percentiles, respectively, the lower and upper whiskers represent the minimum and maximum data points within 1.5 times the interquartile range, and individual data points outside the whiskers represent points exceeding 1.5 times the interquartile ranges.

**Figure 5.**
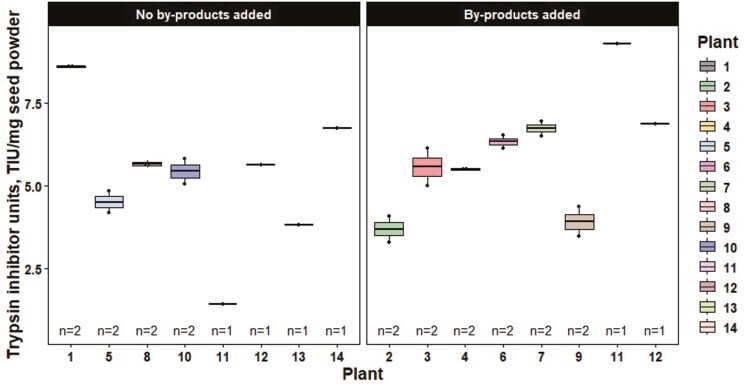
Trypsin inhibitor units of soybean meal samples by soybean processing plant and soybean by-product inclusion (as-is). Individual boxplots are presented for each soybean processing plant where each data point represents a sample analyzed in singlet. The line within the boxplot represents the median, the lower and upper bounds represent the 25th and 75th percentiles, respectively, the lower and upper whiskers represent the minimum and maximum data points within 1.5 times the interquartile range, and individual data points outside the whiskers represent points exceeding 1.5 times the interquartile ranges.

The analytical values for the soybean by-product and soybean meal samples are reported in Tables 1 and 2, respectively. For acid hydrolyzed fat (as-is; Figure 2) and moisture and volatile matter (as-is; Figure 3) content, there was considerable variation regardless of soybean by-product type among processing plants. Similarly, when examining the effect of soybean processing plants on soybean meal composition, there was considerable variation in ether extract (as-is). However, a large portion of the variation in ether extract was due to variation in soybean by-product inclusion (Figure 4). TIU also varied considerably among plants with values ranging from 1.45 to 9.26 TIU/mg of seed powder (as-is; Figure 5).

**Table 1.** Soybean by-product lipid quality analysis by by-product type and sampling timeframe (as-is)^1^^,^^2^

	Timeframe 1^3^				Timeframe 2			
	Gums		Soapstocks		Gums		Soapstocks	
Item	Mean	Range^4^	Mean	Range	Mean	Range	Mean	Range
Moisture and volatile matter, %	17.43	0.15 to 48.79	31.38	12.17 to 60.42	17.90	0.89 to 60.63	25.75	2.99 to 51.40
Insoluble impurities, %	0.26	0.05 to 0.86	2.85	0.14 to 6.82	1.60	0.09 to 6.84	14.42	0.00 to 46.78
Unsaponifiable matter, %	0.55	0.26 to 0.85	0.50	0.28 to 0.95	0.58	0.30 to 0.79	0.61	0.07 to 1.06
P, %	1.10	0.42 to 1.60	0.59	0.16 to 1.03	1.04	0.52 to 1.56	1.16	0.07 to 2.29
p-Anisidine value, %	22.9	1.3 to 58.9	2.1	0.0 to 4.1	3.3	0.0 to 19.4	2.4	0.0 to 4.6
Fat by acid hydrolysis, %	46.48	19.00 to 74.03	22.72	6.85 to 37.87	46.95	20.07 to 72.94	29.09	0.77 to 52.70
SFA, %^5^								
C16:0	14.76	10.53 to 16.27	14.22	11.97 to 16.25	14.51	11.31 to 15.79	13.92	9.52 to 17.23
C17:0	0.12	0.10 to 0.14	0.09	0.00 to 0.15	0.11	0.00 to 0.14	0.10	0.00 to 0.14
C18:0	4.26	3.81 to 4.84	4.40	3.35 to 5.16	4.22	3.55 to 5.17	4.15	3.57 to 4.82
C20:0	0.22	0.18 to 0.27	0.18	0.00 to 0.36	0.23	0.15 to 0.37	0.20	0.00 to 0.32
C22:0	0.41	0.37 to 0.52	0.48	0.37 to 0.74	0.45	0.35 to 0.92	0.37	0.00 to 0.50
C24:0	0.24	0.20 to 0.33	0.32	0.18 to 0.59	0.28	0.21 to 0.67	0.24	0.00 to 0.38
Total SFA	20.05	15.68 to 22.15	19.82	17.93 to 22.06	19.89	15.80 to 22.20	20.20	15.69 to 25.27
MUFA, %^5^								
C16:1	0.08	0.00 to 0.18	0.08	0.00 to 0.18	0.20	0.00 to 0.29	0.20	0.00 to 0.29
C18:1^6^	13.91	10.24 to 18.26	15.75	10.93 to 20.22	15.76	13.23 to 20.17	21.90	13.07 to 52.90
C20:1^6^	0.09	0.00 to 0.18	0.10	0.00 to 0.22	0.21	0.14 to 0.52	0.18	0.00 to 0.29
Total MUFA	14.11	10.34 to 18.49	15.93	10.93 to 20.48	16.22	13.69 to 20.66	22.31	13.61 to 52.90
PUFA, %^5^								
C18:2^6^	57.19	54.89 to 62.43	55.86	52.87 to 63.27	56.89	53.16 to 62.94	50.52	25.00 to 59.20
C18:3^6^	7.68	6.86 to 8.37	7.73	7.33 to 8.00	7.00	5.39 to 7.83	6.97	3.57 to 9.28
Total PUFA	64.87	61.75 to 69.94	63.58	60.74 to 71.14	63.90	60.11 to 69.29	57.50	28.57 to 67.22
Free fatty acids, %^5^	9.69	3.14 to 15.91	7.10	0.16 to 21.40	10.14	6.20 to 33.00	7.58	2.40 to 20.80

^1^A total of 36 soybean by-product samples from 14 soybean processing plants across 8 different companies were tested with 12 soybean gums and 6 soybean soapstocks submitted at each sampling timeframe.

^2^All samples were analyzed in duplicate.

^3^Samples were collected at 2 timeframes within the survey: Timeframe 1 from May to July 2023 and Timeframe 2 from October to November 2023.

^4^The range is the minimum and maximum value for each analytical criterion within soybean by-product type and timeframe of sample collection. Values represent an individual analysis and are not representative of the criteria in duplicate.

^5^Presented as a percentage of extracted lipid.

^6^Concentration includes isomers.

MUFA, monounsaturated fatty acids; PUFA, polyunsaturated fatty acids; SFA, saturated fatty acids.

**Table 2.** Soybean meal analysis by by-product inclusion and sampling timeframe (DM basis)^1^^,^^2^

	Timeframe 1^3^				Timeframe 2			
	No by-products added		By-products added		No by-products added		By-products added	
Item	Mean^4^	Range^5^	Mean	Range	Mean	Range	Mean	Range
DM, %	87.24	85.80 to 88.19	87.73	86.93 to 88.68	87.40	86.69 to 88.48	87.22	86.28 to 88.14
Crude protein, %	53.22 (46.43)	50.98 to 56.33	53.33 (46.79)	51.47 to 55.00	53.28 (46.57)	52.47 to 55.17	52.89 (46.14)	51.07 to 54.01
Ether extract, %	1.15 (1.00)	0.91 to 1.60	1.95 (1.72)	1.17 to 3.35	1.03 (0.90)	0.84 to 1.31	1.56 (1.36)	1.02 to 2.20
Crude fiber, %	5.99 (5.23)	4.01 to 7.39	5.89 (5.16)	4.41 to 8.35	4.58 (4.00)	3.53 to 5.26	4.87 (4.25)	3.95 to 6.15
Ash, %	6.62 (5.77)	6.05 to 7.98	6.65 (5.83)	6.02 to 8.13	6.63 (5.79)	6.23 to 7.09	6.68 (5.83)	6.24 to 7.27
Neutral detergent fiber, %	9.04 (7.88)	6.20 to 12.90	8.22 (7.21)	5.36 to 10.57	7.40 (6.47)	6.18 to 8.92	8.08 (7.04)	6.36 to 9.91
P, %	0.73 (0.64)	0.68 to 0.81	0.72 (0.63)	0.69 to 0.77	0.78 (0.68)	0.73 to 0.83	0.76 (0.67)	0.69 to 0.81
Ca, %	0.40 (0.35)	0.27 to 0.83	0.65 (0.57)	0.29 to 2.27	0.39 (0.34)	0.25 to 0.64	0.50 (0.44)	0.29 to 0.96
TIU/mg seed powder	6.31 (5.50)	4.33 to 9.81	6.52 (5.72)	3.79 to 10.57	6.30 (5.50)	1.67 to 9.85	6.49 (5.65)	3.94 to 7.98

^1^A total of 26 soybean meal samples were collected. At both timeframes, 7 soybean meal samples contained added by-products and 6 samples did not contain added by-products.

^2^All analyses besides trypsin inhibitor units were run in duplicate. Trypsin inhibitor units were analyzed in triplicate.

^3^Samples were collected at 2 timeframes within the survey: Timeframe 1 from May to July 2023 and Timeframe 2 from October to November 2023.

^4^Analytical results are reported on a DM basis except for DM percentage. Values in parentheses represent the means on an as-is basis.

^5^The range is the minimum and maximum value for each analytical criterion within soybean meal by-product inclusion type and timeframe of sample collection. Values represent an individual analysis and are not representative of the criteria in duplicate.

DM, dry matter; TIU, trypsin inhibitor units.

For the soybean by-product analysis (as-is) across both timeframes, soybean gums had a greater (*P* ≤ 0.05) percentage of acid hydrolyzed fat and p-Anisidine value compared to soybean soapstocks (Table 3). However, soybean soapstocks tended to have a greater (*P* = 0.085) percentage of moisture and volatile matter as well as an increased (*P* = 0.052) concentration of insoluble impurities compared to soybean gums. As a percentage of the extracted fat, soybean soapstocks tended to have increased (*P* = 0.085) concentrations of the fatty acid C18:1 which resulted in a tendency for increased (*P* = 0.085) total monounsaturated fatty acids (MUFA) compared to soybean gums. Inversely, as a percentage of the extracted fat, soybean gums tended to have increased (*P* = 0.056) concentrations of the fatty acid C18:2 which translated into a tendency for increased (*P* = 0.082) total polyunsaturated fatty acids (PUFA) compared to soapstocks.

**Table 3.** Effects of soybean by-product type on lipid quality analysis (as-is)^1^^,^^2^

	Soybean by-product			
Item	Gums	Soapstocks	SEM	*P* =^3^
Moisture and volatile matter, %	17.67	28.57	5.00	0.085
Insoluble impurities, %	0.93	8.63	2.975	0.052
Unsaponifiable matter, %	0.56	0.55	0.055	0.902
P, %	1.07	0.89	0.120	0.242
p-Anisidine value, %	13.09	2.23	3.692	0.021
Fat by acid hydrolysis, %	46.72	25.90	4.457	< 0.001
SFA, %^3^				
C16:0	14.64	14.07	0.525	0.386
C17:0	0.12	0.09	0.012	0.165
C18:0	4.24	4.28	0.125	0.810
C20:0	0.23	0.19	0.025	0.191
C22:0	0.43	0.43	0.037	0.973
C24:0	0.26	0.28	0.030	0.714
Total SFA	19.97	20.01	0.566	0.953
MUFA, %^4^				
C16:1	0.14	0.14	0.020	0.935
C18:1^5^	14.83	18.83	1.834	0.085
C20:1^5^	0.15	0.14	0.022	0.658
Total MUFA	15.16	19.12	1.817	0.085
PUFA, %^4^				
C18:2^5^	57.04	53.19	1.588	0.056
C18:3^5^	7.34	7.35	0.228	0.964
Total PUFA	64.39	60.54	1.750	0.082
Free fatty acids, %^4^	9.91	7.34	1.886	0.267

^1^Samples were collected at 2 timeframes within the survey: Timeframe 1 from May to July 2023 and Timeframe 2 from October to November 2023. All samples were analyzed in duplicate.

^2^A total of 24 soybean gums and 12 soybean soapstocks were analyzed at 2 different timeframes from 14 soybean processing plants across 8 different companies.

^3^All results were considered significant with *P* ≤ 0.05 and marginally significant with *P* ≤ 0.10.

^4^Presented as a percentage of extracted lipid.

^5^Concentration includes isomers.

MUFA, monounsaturated fatty acids; PUFA, polyunsaturated fatty acids; SFA, saturated fatty acids.

For the soybean meal analysis across both timeframes, soybean meal containing added soybean by-products had a 61% or a 0.6% unit increase (*P* < 0.05) in ether extract compared to soybean meal samples not containing added soybean by-products on a DM basis (Table 4). There was no evidence of differences (*P* > 0.10) in any other analytical criteria due to by-product inclusion.

**Table 4.** Effects of soybean by-product inclusion on soybean meal composition, DM basis^1^^,^^2^

	Soybean meal by-product inclusion			
Item	No by-products added^3^	By-products added	SEM	*P* =^4^
DM, %	100 (87.32)	100 (87.48)	0.158	0.472
Crude protein, %	53.25 (46.50)	53.11 (46.46)	0.331	0.764
Ether extract, %	1.09 (0.95)	1.76 (1.54)	0.132	0.001
Crude fiber, %	5.28 (4.61)	5.38 (4.71)	0.226	0.760
Ash, %	6.63 (5.79)	6.65 (5.82)	0.122	0.803
Neutral detergent fiber, %	8.22 (7.18)	8.15 (7.13)	0.268	0.846
P, %	0.76 (0.66)	0.74 (0.65)	0.009	0.198
Ca, %	0.40 (0.35)	0.58 (0.51)	0.108	0.232
TIU/mg seed powder	6.31 (5.51)	6.50 (5.69)	0.565	0.815

^1^Samples were collected at 2 timeframes within the survey: Timeframe 1 from May to July 2023 and Timeframe 2 from October to November 2023. All analyses besides trypsin inhibitor units were run in duplicate. Trypsin inhibitor units were analyzed in triplicate.

^2^A total of 26 soybean meal samples were collected. At both timeframes, 7 soybean meal samples contained added by-products and 6 samples did not contain added by-products.

^3^Analytical results and statistics are reported on a DM basis besides DM percentage, which is on an as-is basis. Values in parentheses represent the model-adjusted means on an as-is basis calculated by utilizing the treatment-analyzed dry matter percentage.

^4^All results were considered significant with *P* ≤ 0.05 and marginally significant with *P* ≤ 0.10.

DM, dry matter; TIU, trypsin inhibitor units.

## Discussion

Despite extensive research amassed on soybean meal quality, there has been limited focus on soybean processing by-products. These soybean processing by-products can include weeds and foreign material, soybean hulls, gums, soapstocks, spent bleaching clays, and deodorizer distillates. Soybean by-products have the potential to impact soybean meal quality because soybean gums and soapstocks may be added back to soybean meal during processing, ultimately influencing the final product composition (Gerde et al., 2020). Studies have assessed the potential value of utilizing soybean gums and soapstocks as feed ingredients in livestock diets (Gaiotto et al., 2000; Bruce et al., 2006; Rojas-Cano et al., 2014; Borsatti et al., 2018). However, these studies often did not characterize the composition of the by-products they were using, creating challenges when comparing literature. Furthermore, past literature has focused on evaluating soybean by-products as an ingredient rather than evaluating their effects on soybean meal composition and quality when added directly back to the meal at the soybean processing plant.

In this industry survey, a total of 36 samples across 14 plants were collected. When categorized, 5 plants produced both soybean gums and soapstocks, 7 plants produced only soybean gums, and 2 plants produced only soybean soapstocks. This indicates that most oil refineries are utilizing a degumming step, with only 2 plants not implementing degumming at the time of sample collection. The degumming step of oil refining is viewed as a “precleaning” step for the oil and may not always be implemented based on the initial quality of the crude oil. However, this survey indicates that a majority of plants are implementing this step within their refining process. Approximately 36% of plants sampled utilized both degumming and caustic refining, generating both soybean gums and soapstocks. Interestingly, 50% of plants participating in the study utilized a degumming step potentially in combination with physical refining, ultimately generating only gums. Unlike caustic refining, physical refining removes free fatty acids through steam injections and does not generate soapstocks (Gharby, 2022). Although not a comprehensive evaluation of all soybean processing plants across the United States, this information gives current insight into soybean processing practices.

As expected, there were inherent differences in the composition of soybean by-products. Most notably, soybean gums had 80% greater acid hydrolyzed fat content than soybean soapstocks on an as-is basis. This indicates that there may be more neutral oil loss during degumming than caustic refining. The quantity of neutral oil loss can be greatly affected by the methods used during refining. For degumming, 4 method types may be used including water, acid, dry, or enzymatic degumming. Traditional methods, such as water degumming, typically have a higher oil loss compared to the lesser implemented, newer practice of degumming through enzymes (Dijkstra, 2010). Degumming is used to help prepare crude oil for either the caustic or physical refining step by removing the phosphatides that negatively impact caustic refining. This improves the efficiency of the caustic refining step, resulting in lower oil loss (Woerfel, 1995).

In addition to hydrolyzed fat content, the fatty acid composition in by-products tended to differ with soybean gums having greater concentrations of PUFA and soybean soapstocks having greater concentrations of MUFA. Although these differences are minimal, they may be responsible for differences in the p-Anisidine values. A p-Anisidine value is one method of analysis that determines the level of secondary oxidation products present at the time of analysis offering a general understanding of the oxidation level of a lipid. Polyunsaturated fatty acids are more readily oxidized than MUFA, leading to increased secondary oxidation products and subsequently an increased p-Anisidine value in the higher-PUFA gums (Tao, 2015). Further investigation would be needed to confirm that the p-Anisidine value is impacted by fatty acid content and not related to aspects of the degumming process itself. Despite these differences, the fatty acid contents of both by-products closely reflect that of soybean oil (Dijkstra, 2016).

Besides differences in fat measurements, soapstocks contained a greater percentage of insoluble impurities and tended to have increased moisture and volatile matter when compared to soybean gums. Insoluble impurities can include substances such as sand and debris, oxidized fatty acids, minerals, and alkali soaps (Eurolab, 2024). Inherently, caustic refining involves the removal of free fatty acids utilizing an alkali soap; therefore, it was expected that soapstocks would contain a greater percentage of insoluble impurities. Additionally, although there was only a tendency for differences in moisture and volatile matter percentage between the 2 by-products, the range in moisture content observed is an interesting takeaway. Both soybean gums and soapstocks had an astounding amount of variation with ranges from 0.15% to 60.63% and 2.99% to 60.42%, respectively. This is important when considering the handling and transportation of by-products as well as for potential inclusion into animal diets.

In addition to by-products, a sample of soybean meal was collected from each plant. A total of 26 soybean meal samples were collected across both timeframes. Seven plants reported adding soybean by-products back to soybean meal and 6 plants did not add by-products back during processing in each timeframe. Two plants changed their procedures between sampling timeframes and therefore are represented in both the no by-products added and the by-products added categories in Figures 4 and 5. Multiple factors can impact whether a plant will add soybean by-products back to soybean meal including if they have other marketing streams for the by-products and the initial incoming soybean quality.

According to the soybean meal analysis, there was an increase in ether extract of soybean meal samples when soybean by-products were added back during processing. Woerfel (1995) suggested that the fat content of soybean meal increases by approximately 0.4 percentage units when soybean soapstocks are added back during processing. In alignment with previous literature, the current survey found a 0.7% unit increase in fat content on a DM basis when soybean by-products, either gums or soapstocks, were added back. More specifically, soybean meal devoid of by-products had an ether extract range from 0.84% to 1.60% while soybean meal containing by-products had an ether extract range from 1.02% to 3.35% on a DM basis. Ultimately, if soybean meal is above approximately 1.60% (DM basis) ether extract, consumers can assume some level of soybean by-products have been added back during processing.

Besides differences in ether extract, there were minimal differences in soybean meal composition. From a monogastric nutrition standpoint, the inclusion of soybean by-products may cause concerns about diluting the crude protein content of soybean meal. However, soybean processors only add by-products back at low levels and not at the cost of meeting crude protein specifications (NOPA, 2015). Furthermore, there may be value to using soybean meal containing added by-products in livestock diets. With the current price of fat, soybean by-products may have the potential to serve as an affordable energy source. Therefore, additional research may be justified in evaluating the use of soybean meal containing added by-products in livestock diets.

An unintentional finding of this study was the level and variation of TIU in the soybean meal samples. TIU was evaluated for all soybean meal samples within this study as it serves as an important pillar of soybean meal quality. In the survey, the average TIU was approximately 5.60 TIU/mg of seed powder (as-is) with 73% of soybean meal samples being above 4 TIU/mg of seed powder. The values found in the present survey were comparable to those that have been reported in the past. Chen et al. (2020) collected 192 samples across the United States and reported an average TIU of approximately 5.81 TIU/mg seed powder. High levels of TIU have been shown to negatively impact swine (Yen et al., 1974) and poultry (Erdaw and Beyene, 2018) performance. Chen et al. (2020) found that pigs fed 16.68 TIU/mg of seed powder had reduced digestibility of amino acids and crude protein compared to pigs fed soybean meal with 4.77 or 8.32 TIU/mg of seed powder. However, different feed processing procedures such as pelleting and extrusion may reduce the level of TIU in the soybean meal due to the application of heat (Vagadia et al., 2017). Therefore, the level of TIU in modern soybean meal may present more of a concern to quality than additions of soybean by-products during processing, depending on feed manufacturing procedures. Ultimately, understanding how to reduce the level of TIU as well as reduce the variation in TIU between soybean meal sources is important for integration into monogastric diets.

Although the soybean meal survey aimed to be as comprehensive as possible, there were inherent limitations to this study. First, the samples collected were limited to a subset of the soybean crush facilities throughout the United States and were restricted to the 2 timeframes in which they were collected. As new soybean processing facilities continue to arise and technologies improve, additional information may be needed to evaluate how the composition of these by-products changes over time. Furthermore, due to the level of information that was obtained from the plants and the number of samples collected, the survey was not able to isolate the differences in soybean meal when soybean gums or soybean soapstocks were added back independently. Therefore, information throughout this study was presented as “soybean meal with by-products added”. Finally, although this survey provides some general insight into how soybean by-products affect soybean meal composition and, in return, diet formulation, additional research focused on animal growth performance and health is needed.

In summary, these data suggest soybean gums have a greater acid hydrolyzed fat content and a lower moisture and volatile matter percentage than soybean soapstocks. Most notably, there was considerable variation in by-product composition among processing plants indicating differences in processing procedures or incoming soybean quality. When soybean by-products were added back to soybean meal, there was an increase in ether extract but no effects on crude protein. Ultimately, soybean meal containing greater than approximately 1.6% ether extract on a DM basis likely contains soybean by-products. These results provide information on the current composition and variation of soybean by-products across various processing plants; however, further information is still needed to evaluate their subsequent impact on livestock diets.

## Abbreviations

DM: dry matter
MUFA: monounsaturated fatty acids
NDF: neutral detergent fiber
PUFA: polyunsaturated fatty acids
SFA: saturated fatty acids
TIU: trypsin inhibitor units
